# Sez6l promotes neuropathic pain via Wnt5a/Ca^2+^ pathways in dorsal root ganglion

**DOI:** 10.3389/fgene.2026.1799301

**Published:** 2026-04-20

**Authors:** Limin Hu, Junjie Chen

**Affiliations:** Department of Anesthesiology, Affiliated Hospital of Nantong University, Nantong, China

**Keywords:** dorsal root ganglion (DRG), Foxo1, neuropathic pain, SEZ6L, Wnt5a

## Abstract

**Background:**

Neuropathic pain (NP) is a prevalent chronic pain disorder that severely impairs the physical and mental health of patients, affecting 6.9%–10% of the general population. The dorsal root ganglion (DRG) is a crucial locus in the pathogenesis of NP. However, the underlying mechanisms by which DRGs contribute to this condition remain incompletely understood.

**Methods:**

High-throughput sequencing data of DRGs was downloaded from the Gene Expression Omnibus (GEO) and integrated for analysis. Differential expression analysis combined with multiple machine learning methods was employed to identify candidate genes associated with NP in DRGs. The spared nerve injury (SNI) model was used to assess gene expression patterns. Small interfering RNA-mediated knockdown of the target gene was performed to evaluate its functional role. Bioinformatics analysis and chromatin immunoprecipitation (ChIP) experiments were conducted to explore the transcriptional regulation of the target gene.

**Results:**

Sez6l was identified as a candidate gene upregulated in DRGs. In the SNI model, Sez6l was significantly upregulated. Knockdown of Sez6l reduced the expression levels of inflammatory cytokines (IL-6, TNF-α, and IL-1β) and alleviated mechanical allodynia and thermal hyperalgesia in SNI mice. Bioinformatics analysis and ChIP experiments suggested that Foxo1 may enhance the transcription and expression of Sez6l. Mechanistically, Sez6l promoted NP by activating the Wnt5a/Ca^2+^ signaling pathway in DRGs.

**Conclusion:**

Our findings suggest that Sez6l, which is transcriptionally regulated by Foxo1, facilitates neuropathic pain through activating the Wnt5a/Ca^2+^ signaling pathway in DRGs.

## Introduction

The International Association for the Study of Pain (IASP) defines neuropathic pain (NP) as a discomfort arising from a lesion or pathological condition affecting the somatosensory nervous system (Treede et al., 2007). The dorsal root ganglion (DRG) serves as the aggregation site for the cell bodies of primary sensory neurons. Following peripheral nerve injury, injury signals and inflammatory cytokines are transmitted to the DRGs, which induces a significant increase in the excitability of sensory neurons within the DRGs and the generation of ectopic discharges, thereby directly initiating peripheral sensitization. Meanwhile, DRG neurons synthesize and release pain-related neuropeptides including CGRP and substance P, as well as inflammatory cytokines such as IL-6, TNF-α, and IL-1β. This establishes a local inflammatory microenvironment that further amplifies nociceptive signals, ultimately triggering core symptoms of NP including mechanical allodynia and thermal hyperalgesia, and completing the pathological initiation of pain (Jensen and Finnerup, 2014; Sandkühler, 2009; Campbell and Meyer, 2006).

In addition, the DRGs act as a core pillar for the chronicity of NP through the persistent input of abnormal signals and the disruption of local homeostatic balance (Krames, 2014a). Firstly, ectopic discharges and the release of algogenic transmitters from DRG neurons generate persistent nociceptive inputs to the spinal dorsal horn, inducing synaptic plasticity in spinal neurons and the activation of glial cells, which initiates and maintains central sensitization and results in the sustained amplification of pain signals at the central level (Wu et al., 2025). Secondly, a reciprocally activated positive feedback loop is formed among neurons, satellite glial cells and immune cells within the DRGs, leading to the persistent overexpression of pronociceptive genes and long-term inflammatory responses, rendering peripheral sensitization difficult to reverse spontaneously (Hanani and Spray, 2020; Jager et al., 2020). Finally, the aberrant activation of key signaling pathways such as the Wnt5a/Ca^2+^ pathway in the DRGs establishes a stable pathological signaling axis, which further stabilizes the abnormal neuronal excitability and ultimately drives the progression of NP from acute pain to intractable chronic pain (Zhang et al., 2013). In summary, the DRGs function as the core initiator of peripheral sensitization, the upstream driver of central sensitization and the fundamental basis for the maintenance of chronic pain in NP (Costigan et al., 2009; Kuner and Flor, 2017). The structural and functional remodeling of the DRGs runs through the entire process of the occurrence, development and chronicity of NP (Krames, 2014b).

The Sez6 (Seizure-related homolog protein 6) family, consisting of three transmembrane glycoproteins (Sez6, Sez6l (Sez6-like), Sez6l2 (Sez6-like 2)) predominantly expressed in the central nervous system, has emerged as a key regulatory network in pain research by virtue of its multifaceted regulatory roles in neuronal function, synaptic plasticity, and neuro-immune crosstalk (Roitman et al., 2019; Qiu et al., 2021). At the peripheral level, Sez6 modulates the cell surface trafficking and glycosylation of kainate receptors (KAR) in DRG neurons, while Sez6l is possibly involved in regulating neuronal excitability via the Wnt5a/Ca^2+^ signaling axis—both of which are critical to the induction of peripheral sensitization (Li et al., 2021; McQuate et al., 2017; Gunnersen et al., 2007). Sez6l2 further modulates the local inflammatory microenvironment in the DRGs (Staudt et al., 2016). At the central level, the Sez6 family regulates synaptic plasticity in the spinal dorsal horn, thereby sustaining central sensitization and the chronicity of pain (Gunnersen et al., 2007). Recent studies employing knockout/knockin mouse models have confirmed the regulatory role of the Sez6 family in NP models, highlighting its potential as a diagnostic biomarker (Nash et al., 2020). As research progresses, the integration of single-cell omics, organoid models, and the development of specific inhibitors will enable the Sez6 family to provide new insights into the mechanisms and treatment of chronic pain.

Forkhead box O1 (Foxo1) is one of the core members of the Foxo family (Maiese, 2021). As a multifunctional transcription factor, it is involved in various cellular functions, including cell cycle regulation, DNA damage repair, apoptosis, metabolism, stress response, and cell differentiation. Foxo1 directly binds to the voltage-gated sodium channel Nav1.7, increases current density, enhances neuronal excitability, and promotes the occurrence of mechanical allodynia (Zhang et al., 2021). It can also regulate the expression of neurotrophic factor receptors and inflammation-related genes, participate in post-injury neuronal plasticity, and facilitate peripheral sensitization (Zhang et al., 2018). Additionally, Foxo1 promotes the activation of glial cells, inducing them to release a large number of proinflammatory cytokines (IL-1β, TNF-α, IL-6) and chemokines, which amplify pain signals and maintain central sensitization (Hossain et al., 2017; Reinhold et al., 2021). Foxo1 reduces the autophagic activity of neurons and glial cells, exacerbating the chronicity of pain (Ali et al., 2020). Overall, Foxo1 plays a crucial role in the pathophysiological process of NP and exhibits a high degree of overlap with the Sez6 family in terms of tissue distribution, signaling pathways, and functional regulation (Zhang et al., 2021; Pigoni et al., 2020). Currently, the cross-regulatory mechanisms between the two in NP have not been fully elucidated. In-depth exploration of the synergistic effect between Foxo1 and the Sez6 family will provide important theoretical basis and new research perspectives for improving the etiological theory of NP and developing novel precise analgesic strategies.

The Wnt5a/Ca^2+^ pathway, a subtype of the non-canonical Wnt signaling pathway, is characterized by Wnt5a as its specific ligand and abnormal elevation of intracellular free Ca^2+^ concentration (McQuate et al., 2017; Akoumianakis et al., 2022). Independent of the canonical β-catenin-dependent transcriptional pathway, this pathway has emerged as a key signaling axis governing the occurrence and progression of NP (McQuate et al., 2017; Simonetti and Kuner, 2020). Specifically, it plays a central initiating role in peripheral sensitization: following peripheral nerve injury, Wnt5a expression is significantly upregulated, which mediates the release of calcium from the endoplasmic reticulum in DRG neurons, thereby enhancing neuronal excitability and inducing ectopic discharges (Xie et al., 2022; Zhai et al., 2024).

Although the role of the Wnt5a/Ca^2+^ pathway in NP has been initially verified, the specific molecular details underlying its synergistic regulation of NP together with Sez6l and Foxo1 remain poorly understood. In-depth exploration of the regulatory mechanisms of the Wnt5a/Ca^2+^ pathway in NP, as well as the clarification of its molecular targets as a core signaling hub, will provide crucial theoretical support and research directions for refining the etiological theory of NP.

## Methods

### Data acquisition and preprocessing

High-throughput sequencing datasets of mouse dorsal root ganglion (DRG) were retrieved from the Gene Expression Omnibus (GEO) database (https://www.ncbi.nlm.nih.gov/geo/). The GSE89224 and GSE102721 datasets were selected based on the following inclusion criteria: (i) samples derived from the SNI NP model; (ii) availability of both sham-operated controls and SNI groups. After integration, the combined dataset comprised 10 sham-operated and 6 SNI DRG samples. To eliminate batch effects arising from different sequencing platforms and experimental conditions, ComBat-seq (sva R package) was applied to the raw count matrix with empirical Bayes shrinkage adjustment. The effectiveness of batch correction was evaluated by Principal Component Analysis (PCA) using the prcomp function in R (version 4.2.1) based on the top 500 most variable genes (selected by median absolute deviation).

### Least absolute shrinkage and selection operator (LASSO) analysis

To identify the most informative genes associated with NP, LASSO logistic regression with 10-fold cross-validation was implemented using the glmnet package (version 4.1–6). The optimal tuning parameter (lambda, λ) was determined by the minimum criteria (lambda.min) and 1-standard error criteria (lambda.1se) based on binomial deviance. The LASSO model identified seven non-zero coefficient features as candidate biomarkers. Coefficient profiles were plotted against log(lambda) to illustrate the regularization path.

### Support vector machine (SVM) analysis

Recursive feature elimination (RFE) with SVM (radial basis function kernel) was performed using the caret package (version 6.0–92) to rank genes by their importance in classifying Sham vs. SNI samples. The optimal number of features was determined by evaluating classification accuracy (maximized at 6 genes) and error rate (minimized at 6 genes) across iterative subset selection.

### Cell culture

The HEK-293T cell line was obtained from the Shanghai Institutes for Biological Sciences (Shanghai, China). Cells were cultured in DMEM supplemented with 10% fetal bovine serum and 1% penicillin-streptomycin (Pricella, China).

### Isolation and culture of DRGs

Following euthanasia, mice were placed in the prone position and the dorsal skin disinfected with 70% ethanol. A midline skin incision was performed from the thoracic to the sacral region, and the paravertebral muscles were bluntly dissected to expose the lumbar vertebrae (L4–L6). Under microscopic visualization, the vertebral laminae were carefully removed unilaterally to expose the spinal cord and associated nerve roots. DRGs, visualized as bilaterally symmetric, whitish oval swellings situated at the intervertebral foramina, were gently excised using fine forceps by severing the attached dorsal and ventral roots, and immediately immersed in ice-cold PBS. Dissociated DRGs were cultured in Neurobasal Medium (Gibco) supplemented with 10% fetal bovine serum, 5mL L-glutamine (200mM) (Gibco, USA), 10mL B-27 Supplement(50x) (Gibco, USA), 5ml Penicillin-Streptomycin(100X) (Pricella, China).

### Intracellular transfection and intrathecal injection of adeno-associated virus

For intracellular transfection, vectors or shRNA (100nM; transfected with Lipo3000) was added directly to DRGs cultured in 6-well plates, and the cells were collected after 3 days. For intrathecal Injection, a polyethylene-10 catheter was inserted into the subarachnoid space of mice, and 10 mL of the virus was administered into the cerebrospinal fluid. The control group received an equivalent amount of control adeno-associated virus. Experimental procedures were carried out 21 days post-injection.

The sequences of shRNAs.

**Table:** 

Target	Sequences
Sez6l	shRNA1 5′-CGGGACCTTCCAGCTACATTA-3′ shRNA2 5′-GAGTACTTAAAGCCCATATTT-3′
Foxo1	shRNA 5′-CGG​AGG​ATT​GAA​CCA​GTA​TAA-3′
Foxo3	shRNA 5′-CGG​CAC​CAT​GAA​TCT​GAA​TGA-3′
Vdr	shRNA 5′- TTA​AAT​GTG​ATT​GAT​CTC​AGG-3′
SOX10	shRNA 5′-CAG​CCC​TCA​GGA​CCC​TAT​TAT-3′
Wnt5a	shRNA 5′-CCA​CTT​GTA​TCA​GGA​CCA​CAT-3′

### Quantitative reverse transcription-polymerase chain reaction (qRT-PCR)

Total RNA was extracted from mouse DRGs using TRIzol reagent (Thermo Fisher Scientific, USA) according to the manufacturer’s protocol. RNA concentration and purity were assessed by NanoDrop spectrophotometry (A260/A280 ratio 1.8–2.0). First-strand cDNA was synthesized using the SuperScript™ IV Reverse Transcriptase kit (Invitrogen, USA) with oligo(dT) primers. The cDNA product was diluted 1:5 with nuclease-free water (100–200 ng/μL), and 2 μL was used as template per qPCR reaction. qRT-PCR was performed in 96-well plates using SYBR Green PCR Master Mix (Applied Biosystems, USA) on a QuantStudio 5 Real-Time PCR System (Thermo Fisher). Each 20 μL reaction contained: 10 μL of 2× SYBR Green Master Mix, 0.8 μL of forward primer (10 μM), 0.8 μL of reverse primer (10 μM), 2 μL of diluted cDNA template (∼200–400 ng), and 6.4 μL of RNase-free water. The thermal cycling conditions were as follows: initial denaturation at 95 °C for 10 min; followed by 40 cycles of denaturation at 95 °C for 15 s, annealing at 60 °C for 30 s, and extension at 72 °C for 30 s; with a final extension at 72 °C for 5 min. Melting curve analysis (60 °C–95 °C) was performed to verify product specificity. Relative gene expression was normalized to β-actin (internal control) and calculated using the 2^−ΔΔCt^ method. All reactions were performed in triplicate.

The sequences of primers.

**Table:** 

Target	Sequences
Sez6l	Forward (5‘-3’) -GTATCCGCATTGAGTTCACGTCG Reverse (5‘-3’) -GTGAACTCCACAATGGTGCCGA
Foxo1	Forward (5‘-3’) -AGATGAGTGCCCTGGGCAGC Reverse (5‘-3’) -GATGGACTCCATGTCACAGT
β-actin	Forward (5‘-3’) -AAATCGTGCGTGACATCAAAGA Reverse (5‘-3’) -GCCATCTCCTGCTCGAAGTC
Atf3	Forward (5‘-3’) -ATAAACACCTCTGCCATCGG Reverse (5‘-3’) -GCCTCCTTTTCCTCTCATCTTC
Gpr151	Forward (5‘-3’) -AAGGGCGTTTGGGATCTCG Reverse (5‘-3’) -GCTTTGGCTACCACAACAAATGT
Vdr	Forward (5‘-3’) -ACCCTGGTGACTTTGACCG Reverse (5‘-3’) -GGCAATCTCCATTGAAGGGG
SOX10	Forward (5‘-3’) -TCAGACGAACAAGGCTGTCC Reverse (5‘-3’) -CCATCTAGGCAATCTCGGTCTC
Foxo3	Forward (5‘-3’) -TACGAGTGGATGGTGCGCTGT Reverse (5‘-3’) -TCATTCTGAACGCGCATGAAGC

### Western blotting

Protein lysates were prepared from mouse DRGs using RIPA lysis buffer (Beyotime, China) supplemented with 1% protease inhibitor cocktail (Roche, Switzerland) and 1% phosphatase inhibitors. Protein concentrations were determined using the BCA Protein Assay Kit (Thermo Fisher Scientific, USA) according to the manufacturer’s protocol. Equal amounts of protein (30 μg per lane) were separated on 10% SDS-PAGE gels at 80 V for 30 min followed by 120 V for 60–90 min. Proteins were then transferred onto 0.22 μm PVDF membranes (Millipore, USA) using a wet transfer system at 300 mA constant current for 90 min at 4 °C in transfer buffer (25 mM Tris, 192 mM glycine, 20% methanol). After transfer, membranes were blocked with 5% non-fat dry milk in TBST (Tris-buffered saline with 0.1% Tween20) for 1 h at room temperature. The membranes were then incubated with primary antibodies overnight at 4 °C at the following dilutions: Sez6l (1:1000, Thermo Fisher Scientific, PA5-143994), β-actin (1:5000, Proteintech, 66009-1-Ig), Foxo1 (1:1000, Proteintech, 18592-1-AP), Frizzled2 (1:800, Proteintech, 24272-1-AP), CaMKII (1:1000, Cell Signaling Technology, 4436), p-CaMKII (Thr286) (1:500, Cell Signaling Technology, 12,716), and Wnt5a (1:1000, Cell Signaling Technology, 2392).After washing three times with TBST (10 min each), membranes were incubated with horseradish peroxidase (HRP)-conjugated secondary antibodies (goat anti-rabbit/mouse IgG (1:5000), Proteintech, China) for 1–2 h at room temperature. Following three additional washes with TBST, protein bands were visualized using enhanced chemiluminescence substrate (Millipore, USA) and captured using a ChemiDoc XRS + imaging system (Bio-Rad, USA). Band intensities were quantified using ImageJ software and normalized to β-actin loading controls.

### Animals

Adult male C57BL/6J mice (6–8 weeks old, 20–25 g) were purchased from the Experimental Animal Center of GemPharmatech (Jiangsu, China). Upon arrival, mice were housed in specific pathogen-free (SPF) conditions under a controlled environment with a 12-h light/dark cycle (lights on at 08:00), constant temperature (22 °C ± 2 °C), and relative humidity (50%–60%). Animals were group-housed (3–5 mice per cage) in individually ventilated cages (IVC) with corn cob bedding and provided with *ad libitum* access to standard rodent chow and filtered tap water. All mice were allowed to acclimate to the facility for at least 7 days prior to experimental procedures. All animal protocols were approved by the Institutional Animal Care and Use Committee (IACUC) of Nantong University and performed in accordance with the National Institutes of Health Guide for the Care and Use of Laboratory Animals.

Mice were randomly assigned to experimental groups using a computer-generated randomization sequence. The investigators performing behavioral tests and data analysis were blinded to group allocation. n = 4 mice per group for behavioral experiments. All experiments were performed with at least three independent biological replicates.

### Spared nerve injury (SNI) model

The protocol for SNI model was performed as previously reported (Chaplan et al., 1994). Behavioral testing was performed at baseline (day 0) and on days 1, 3, 6, and 9 post-surgery. Intrathecal injections of adeno-associated virus were administered 7 days after SNI surgery, and subsequent experiments were performed 21 days post-injection (28 days post-SNI). Briefly, place C57BL/6J mice under appropriate anesthesia and perform disinfection and aseptic procedures on the mid-thigh region. Under a microscope, expose the sciatic nerve through an incision and identify its three main branches: the tibial nerve, the common fibular nerve, and the sural nerve. Using non-absorbable suture, ligate the common fibular nerve and the sural nerve branches while preserving the integrity of the tibial nerve. Upon completion of the procedure, suture the surgical site and administer suitable analgesics and antibiotics. Allow the animals to recover in a warm and comfortable environment.

### Enzyme-linked immunosorbent assay (ELISA)

ELISA kits for IL-1β, IL-6, and TNF-α were purchased from Sangon Biotech (Shanghai, China). The expression levels of these cytokines in DRG samples were measured according to the manufacturer’s instructions. The experimental procedure was performed as follows: (a) Add 100 μL of standard working solution and detection samples to each well. Standards were prepared in duplicate. The plate was sealed and incubated at 37 °C for 90 min. (b) Discard the liquid, tap dry, and add 100 μL of biotin-labeled antibody working solution to each well. The plate was sealed and incubated at 37 °C for 60 min. (c) Discard the liquid, tap dry, and add 350 μL of wash solution to each well. Incubate for 1–2 min, then tap dry. Repeat this step 4 times. (d) Add 100 μL of HRP-labeled streptavidin working solution to each well. The plate was sealed and incubated at 37 °C for 30 min. (e) Add 300 μL of wash solution to each well, incubate for 30 s, and tap dry. Repeat this step 4 times. Add 90 μL of chromogenic reagent (protected from light) to each well. The plate was sealed and incubated in the dark at 37 °C for approximately 15 min. (f) Add 50 μL of stop solution to each well, and immediately measure the optical density at 450 nm using an enzyme immunoassay analyzer (within 5 min).

### Behavioral testing

Mechanical allodynia and thermal hyperalgesia were assessed using the paw withdrawal threshold (PWT) and paw withdrawal latency (PWL) tests, respectively (Chaplan et al., 1994; Hargreaves et al., 1988). For mechanical allodynia assessment, mice were placed in transparent boxes with a metal mesh floor, and pressure was applied to the plantar surface of the hindpaw using an electric von Frey device. For thermal hyperalgesia assessment, the PWL test was conducted using a plantar test instrument. Briefly, thermal stimuli were applied to the paw, and the withdrawal latency was recorded and analyzed, with a cut-off time of 30 s to prevent tissue damage.

### Chromatin immunoprecipitation (ChIP)

ChIP experiments were performed using a ChIP Assay Kit (Beyotime, Shanghai, China) according to the manufacturer’s protocol with minor modifications. Briefly, cells were cross-linked with 1% formaldehyde for 10 min at room temperature and quenched with 0.125 M glycine for 5 min. After washing twice with ice-cold PBS, cells were resuspended in ChIP Sonication Buffer supplemented with 1× protease inhibitor cocktail and lysed on ice for 10 min. Chromatin was sheared by sonication to generate DNA fragments of 200–1000 bp. The lysate was centrifuged at 12,000–14,000 × g for 5 min at 4 °C. Approximately 200 μL of the supernatant was transferred to a fresh 2 mL centrifuge tube and maintained on ice. For immunoprecipitation, chromatin was incubated with 2 μg of anti-Foxo1 antibody (Proteintech, 18592-1-AP) or normal rabbit IgG (negative control) overnight at 4 °C with rotation. Subsequently, 40 μL of Protein A/G MagPoly Beads (Beyotime) were added and incubated for 1–2 h at 4 °C. The bead-antibody complexes were sequentially washed with low-salt wash buffer, high-salt wash buffer, LiCl wash buffer, and TE buffer (two washes per buffer, 5 min each). Immunoprecipitated chromatin was eluted in elution buffer and reverse cross-linked at 65 °C for 4–5 h following treatment with RNase A (37 °C, 30 min) and proteinase K (45 °C, 1–2 h). DNA was purified using spin columns provided in the kit and eluted in 30–50 μL of nuclease-free water. For PCR detection, purified ChIP DNA was analyzed by quantitative real-time PCR using SYBR Green qPCR Mix (Beyotime) and specific primers targeting the promoter regions of interest. Relative enrichment was calculated using the 2^(-ΔΔCt)^ method and normalized to input DNA.

The sequences of CHIP-PCR primers: F:5′-CCTGGGGCTGTGCTACTATG -3’; R: 5′-ACA​TCA​GCA​ATT​TGT​GTG​TCT​T -3’.

### Luciferase reporter assay

Luciferase reporter vectors containing the wild-type and mutated fragments of Sez6l promoter were constructed by RiboBio (Guangzhou, China). The promoter region of Sez6l containing the putative Foxo1 binding sites was amplified from genomic DNA using specific primers flanked by *Kpn*I and *Hin*dIII restriction sites. The PCR product was cloned into the pGL3-Basic vector upstream of the firefly luciferase gene to generate the wild-type or mutant Sez6l promoter reporter. Luciferase assay was performed by using Luciferase Reporter Gene Assay Kit (Yeasen, Shanghai). Briefly, the constructed vector was transfected into 293T cells, followed by incubation on ice for 5 min after the addition of lysis buffer. Next, 20 μL of lysate was transferred into a measurement tube. Subsequently, 100 μL of luciferase assay reagent was added, and the tube was mixed on a shaker 2–3 times for thorough mixing. Relative light units (RLU) were then measured after adequate mixing.

### Statistical analysis

All statistical analyses were performed with GraphPad Prism 9.0 software (USA). The Shapiro-Wilk test was utilized to measure data normality. Repeated measures two-way analysis of variance with Tukey’s post hoc tests were used to analyze behavioral data. For qRT-PCR, Western blot, ELISA, ChIP, and luciferase reporter assays, one-way ANOVA with Tukey’s post hoc test was used for multiple group comparisons, and unpaired two-tailed Student’s t-test was used for two-group comparisons. All experiments were performed with at least three independent biological replicates. Data are presented as mean ± SEM. A value of P < 0.05 was considered statistically significant.

## Results

### Utilization of SNI-induced NP databases

**FIGURE 1:**
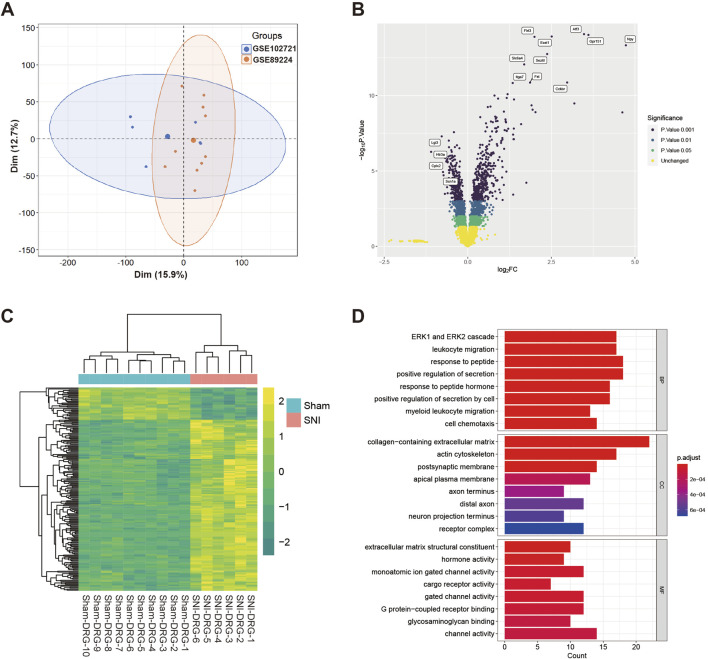
Principal Component Analysis (PCA) and Differential Gene Expression Analysis **(A)** PCA results of the 16 samples showing a centralized data distribution. **(B)** Volcano plot illustrating the differentially expressed genes (DEGs) between the Sham and SNI groups. **(C)** Heatmap representation of the DEGs between the Sham and SNI groups. **(D)** Gene Ontology (GO) enrichment analysis of DEGs.

First, we obtained 10 sham-operated and 6 SNI DRG samples from the GSE89224 and GSE102721 datasets. ComBat-seq was subsequently applied to eliminate batch effects between the datasets. Principal component analysis (PCA) showed that the data distribution of all 16 samples was relatively centralized, indicating that the merged dataset was reliable (Figure 1A). Differential gene expression analysis identified 170 upregulated and 32 downregulated genes between the sham and SNI groups, as shown in the volcano plot and heatmap (Figures 1B,C). Gene Ontology (GO) enrichment analysis revealed that differentially expressed genes (DEGs) were involved in biological processes (BP), cellular components (CC), and molecular functions (MF) (Figure 1D). The major alterations in BP included the ERK1 and ERK2 cascade, leukocyte migration, and response to peptide. The primary changes in CC were collagen-containing extracellular matrix, actin cytoskeleton, and postsynaptic membrane. The main variations in MF were extracellular matrix structural constituent, hormone activity, and gated channel activity.

### Identification of Sez6l in DRGs of SNI model

**FIGURE 2:**
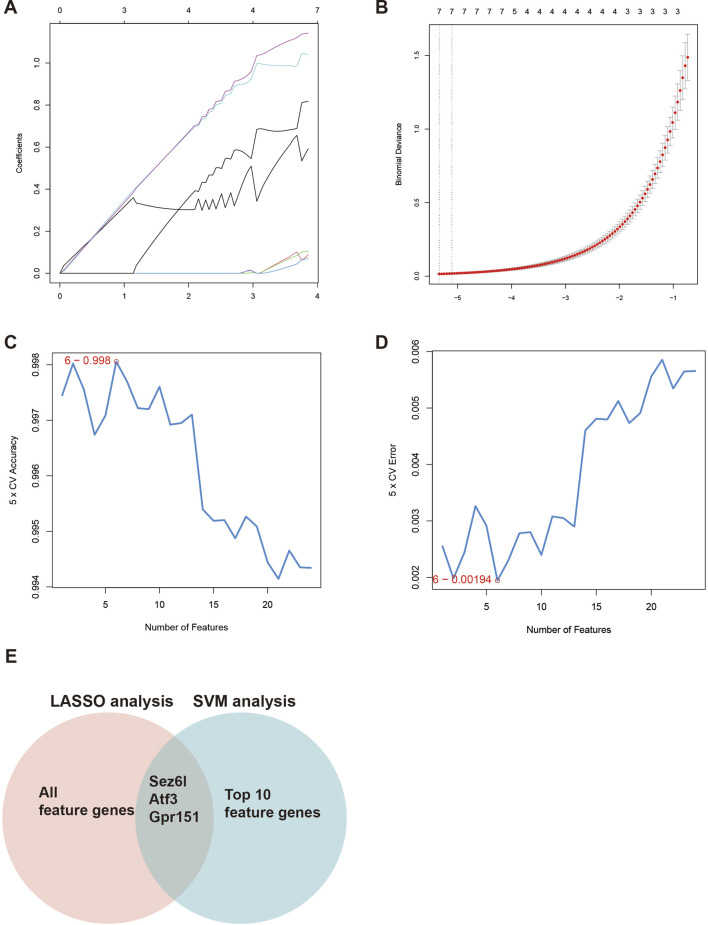
Identification of key dysregulated genes in DRGs of the SNI model using machine learning approaches. **(A)** LASSO regression analysis of DEGs identified seven genes as significant features. **(B)** LASSO coefficient profiles of the seven feature genes selected by the optimal penalty parameter. **(C)** SVM accuracy analysis showing that the classification accuracy was maximized with six genes. **(D)** SVM error analysis indicating that the classification error was minimized with six feature genes. **(E)** Venn diagram showing the overlap between LASSO- and SVM-selected genes.

**FIGURE 3:**
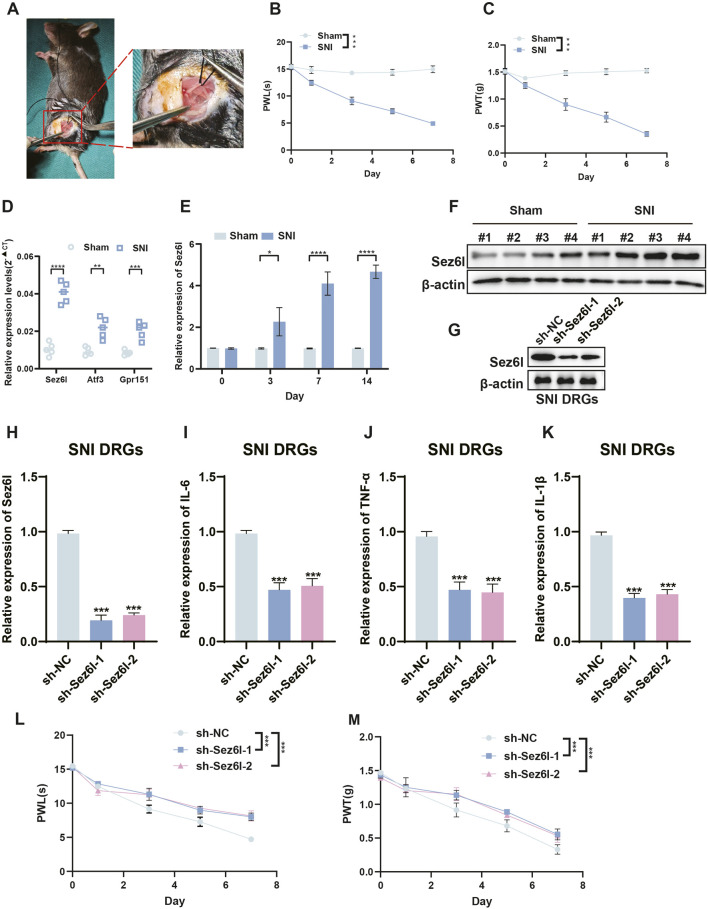
Sez6l upregulation in the SNI mouse model and the effects of Sez6l knockdown on NP. **(A)** Schematic diagram of constructing the SNI model. **(B)** PWL assays showed that paw withdrawal latencies were decreased after construction of SNI model. **(C)** PWT assays showed that paw withdrawal threshold was decreased after construction of SNI model. **(D)** qRT-PCR analysis was used to reveal Sez6l, Atf3 and Gpr151 expression in the DRGs of SNI mice. **(E)** qRT-PCR analysis revealed that Sez6l expression was significantly upregulated in the DRGs of SNI mice. **(F)** Western blot revealed that Sez6l expression was significantly upregulated in the DRGs of SNI mice. **(G)** Western blot analysis of Sez6l expression showing the knockdown efficiency of sh-Sez6l-1 and sh-Sez6l-2 in DRGs. **(H)** qRT-PCR results of Sez6l mRNA levels in the DRGs after knockdown with sh-Sez6l-1 or sh-Sez6l-2. **(I–K)** ELISA analysis of IL-6, TNF-α, and IL-1β expression in the DRGs. **(L)** PWL assays showed that Sez6l knockdown increased paw withdrawal latencies. **(M)** PWT assays showed that Sez6l knockdown increased paw withdrawal threshold. n = 3 biological replicates per group for qRT-PCR and Western blot. n = 4 biological replicates per group for behavioral assays. Statistical analysis was performed using one-way ANOVA with Tukey’s post hoc test for qRT-PCR, Western blot, and ELISA data, and repeated measures two-way ANOVA for behavioral data. All experiments were performed with three independent biological replicates. Data are presented as mean ± SEM. *p < 0.05, **p < 0.01, ***p < 0.001.

To identify the main regulators in DRGs of the SNI model, we analyzed DEGs using two machine learning methods: least absolute shrinkage and selection operator (LASSO) and support vector machine (SVM). LASSO regression identified seven genes as significant features (Figure 2A). The parameters of these seven features in the LASSO regression model are shown in Figure 2B. SVM-accuracy analysis showed that classification accuracy was maximized when the number of feature genes was six. Additionally, SVM error analysis indicated that the error value was minimized when the number of feature genes was also six (Figures 2C,D). Three candidate genes (Sez6l, Atf3, and Gpr151) were finally selected by combining LASSO and SVM analysis (Figure 2E). Among the screened genes, Sez6l has been reported as a BACE1 substrate and to participate in movement disorders and neuropsychiatric diseases. We successfully established the SNI mouse model (Figures 3A–C; Supplementary Figure S1B). Moreover, qRT-PCR was performed to quantify the expression levels of the above genes in the SNI and sham groups. Sez6l showed the most significant difference in expression level among the three genes (Figure 3D). However, the role of Sez6l in NP remains poorly understood, necessitating further investigation.

### Sez6l knockdown represses SNI-induced NP

Subsequently, Western blot and qRT-PCR analysis showed that Sez6l expression was significantly upregulated in the SNI mouse model (Figures 3E,F, Supplementary Figure S1A). To investigate the biological role of Sez6l in NP, we constructed a Sez6l knockdown mouse model by intrathecally injecting sh-NC, sh-Sez6l-1, or sh-Sez6l-2 into SNI mice. Intrathecal injection was performed 1 week after SNI surgery. Western blot and qRT-PCR results showed that protein and mRNA levels of Sez6l in SNI DRGs were significantly decreased after sh-Sez6l-1 or sh-Sez6l-2 injection (Figures 3G,H). Additionally, ELISA results showed that Sez6l knockdown inhibited the expression of NP-associated inflammatory cytokines (IL-6, TNF-α, and IL-1β) in SNI DRGs (Figures 3I–K), but not in sham DRGs (Supplementary Figures S1C-S1E). We then performed NP behavioral tests, including paw withdrawal latency (PWL) and paw withdrawal threshold (PWT) assays. Results showed that Sez6l knockdown increased paw withdrawal thresholds and paw withdrawal latencies (Figures 3L,M). Collectively, these experiments suggested that inhibition of Sez6l relieved SNI-induced NP.

### Forkhead box protein O1 (Foxo1) transcriptionally upregulates the Sez6l expression

**FIGURE 4:**
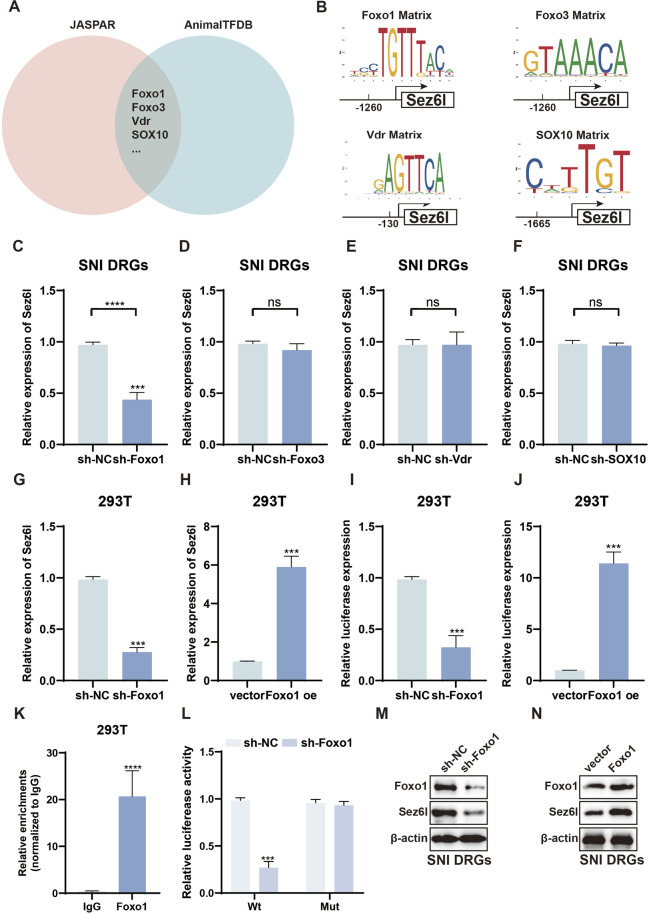
Regulation of Sez6l expression by Foxo1. **(A,B)** Analysis of transcription factors predicted to regulate Sez6l. **(C–F)** qRT-PCR assays show that Sez6l expression is significantly decreased in DRGs transfected with sh-Foxo1. **(G–J)** Sez6l luciferase promoter-reporter assays demonstrate that Foxo1 shRNA or overexpression vectors lead to increased or decreased luciferase activity in 293T cells, respectively. **(K)** ChIP assay reveals significant enrichment of the Sez6l promoter region containing the Foxo1 binding site when using Foxo1 antibody, compared to control IgG. **(L)** Luciferase reporter assay was used to detect the Sez6l luciferase promoter activity in 293T cells transfected with sh-Foxo1. **(M–N)** Western blot analysis of Sez6l expression in indicated groups. n = 3 biological replicates per group for qRT-PCR, Western blot, ChIP, and luciferase assays. Statistical analysis was performed using one-way ANOVA with Tukey’s post hoc test or unpaired two-tailed Student’s t-test as appropriate. Data are presented as mean ± SEM. *p < 0.05, **p < 0.01, ***p < 0.001.

Next, we explored the underlying mechanism of Sez6l upregulation. Transcription factors are principal regulators of selective gene expression, directing transcriptional programs *in vivo*. Dysregulation of these programs is a hallmark of pathogenesis. JASPAR (http://jaspar.genereg.net) and the Animal Transcription Factor Database (AnimalTFDB, http://bioinfo.life.hust.edu.cn/AnimalTFDB/) were used to analyze the Sez6l promoter. The analysis showed that twenty-two transcription factors may regulate Sez6l. Among these candidates, SOX10, Foxo1, Foxo3, and Vdr have been reported to participate in the regulation of NP (Figures 4A,B). qRT-PCR analysis showed that Foxo1, Foxo3, and SOX10 expression was enhanced in SNI DRGs (Supplementary Figures S2A). We subsequently used qRT-PCR assays to evaluate the efficiency of shRNAs targeting Foxo1, Foxo3, Vdr, and SOX10 (Supplementary Figures S2B-S2E). Further qRT-PCR assays revealed that Sez6l expression was significantly reduced in SNI DRGs transfected with sh-Foxo1, but not with other shRNAs (Figures 4C–F). qRT-PCR assays revealed that Sez6l expression was significantly downregulated in 293T cells transfected with sh-Foxo1, and markedly upregulated in cells overexpressing Foxo1 (Figures 4G,H). The Sez6l luciferase promoter-reporter assay indicated that luciferase activity was increased or decreased after transfection of Foxo1 shRNA or overexpression vectors in 293T cells (Figures 4I,J). Based on the single predicted Foxo1 binding site in the Sez6l promoter region, we designed specific primers for ChIP assay. ChIP results showed that the fragment containing the binding sites was significantly enriched with Foxo1 antibody compared with control IgG (Figure 4K). Furthermore, we constructed a mutated Sez6l luciferase promoter-reporter. The luciferase reporter assay showed that luciferase activity was decreased or unchanged in wild-type or mutated Sez6l promoter reporter in 293T cells transfected with sh-Foxo1 (Figure 4L). Western blot analysis showed that Sez6l expression was decreased or increased in SNI DRGs transfected with sh-Foxo1 or Foxo1 overexpression vectors, respectively (Figures 4M,N). In all, these data revealed that Foxo1 upregulated Sez6l by enhancing its transcription.

### Foxo1-Sez6l axis regulates SNI-induced NP

**FIGURE 5:**
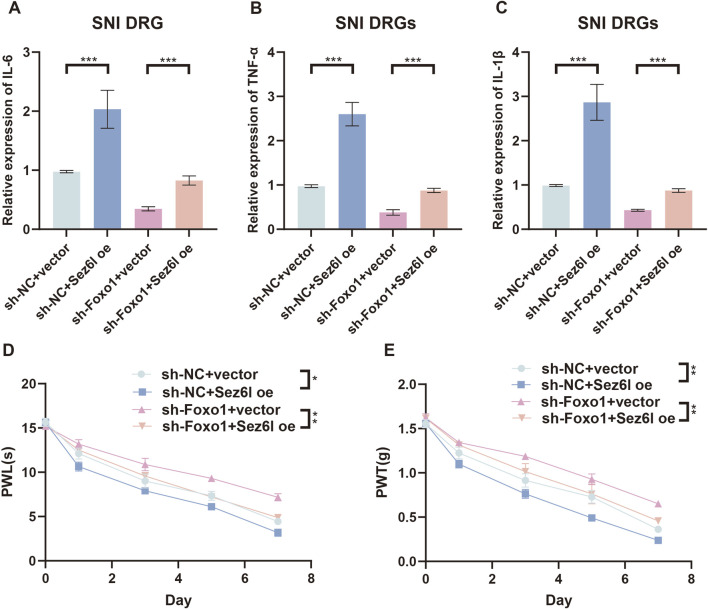
Effects of Foxo1 knockdown and Sez6l overexpression on inflammatory cytokine expression and pain behavior in DRGs. **(A–C)** ELISA assay results showing the expression levels of IL-6, TNF-α, and IL-1β in SNI DRGs transfected with sh-Foxo1 and Sez6l overexpressing vectors. **(D)** PWL assays showed that paw withdrawal latencies were increased by Foxo1 knockdown, while co-transfection of sh-Foxo1 and Sez6l overexpressing vectors rescued these pain behaviors. **(E)** PWT assays showed that paw withdrawal threshold was increased by Foxo1 knockdown, while co-transfection of sh-Foxo1 and Sez6l overexpressing vectors rescued these pain behaviors. n = 3 biological replicates per group for ELISA. n = 4 biological replicates per group for behavioral assays. Statistical analysis was performed using one-way ANOVA with Tukey’s post hoc test for ELISA data and repeated measures two-way ANOVA for behavioral data. Data are presented as mean ± SEM. *p < 0.05, **p < 0.01, ***p < 0.001.

To further elucidate the regulatory role of Foxo1 in NP, rescue experiments were performed *in vivo* and *in vitro*. ELISA results showed that IL-6, TNF-α, and IL-1β expression was decreased in SNI DRGs transfected with sh-Foxo1 but upregulated upon co-transfection with Sez6l overexpression vectors, whereas cytokine levels remained unchanged in sham DRGs under both conditions (Figures 5A–C and S3A-S3C). PWL and PWT assays showed that hyperalgesia and allodynia were attenuated by Foxo1 knockdown but rescued by co-transfection of sh-Foxo1 and Sez6l overexpression vectors (Figures 5D,E). These results showed that the Foxo1-Sez6l axis regulated NP in the SNI model.

### Upregulated Wnt5a/Ca^2+^ signaling pathway promotes SNI-induced NP

**FIGURE 6:**
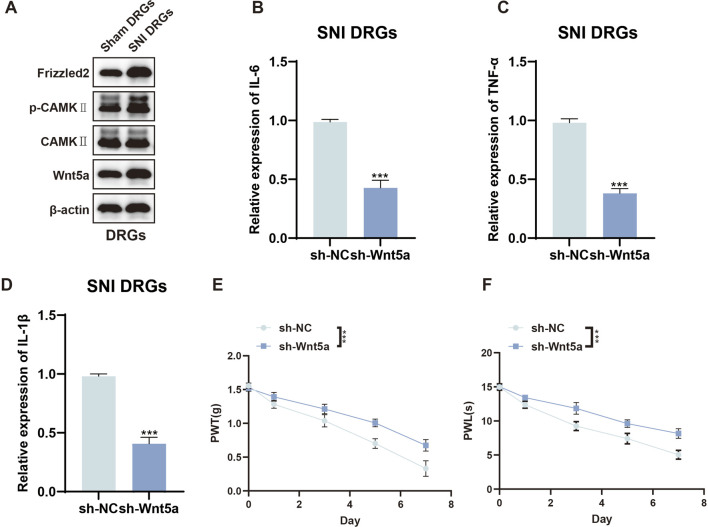
Effects of Wnt5a knockdown on inflammation and pain behavior in SNI mice. **(A)** Western blot analysis of Wnt5a, Frizzled2, and the ratio of p-CAMKIIexpression. **(B–D)** ELISA assay analysis of IL-6, TNF-α, and IL-1β expression in the indicated SNI DRGs. **(E)** PWL assays showed that paw withdrawal latencies were increased by Wnt5a knockdown. **(F)** PWT assays showed that paw withdrawal threshold was increased by Wnt5a knockdown. n = 3 biological replicates per group for ELISA. n = 4 biological replicates per group for behavioral assays. Statistical analysis was performed using one-way ANOVA with Tukey’s post hoc test for Western blot and ELISA data, and repeated measures two-way ANOVA for behavioral data. Data are presented as mean ± SEM; *p < 0.05, **p < 0.01, ***p < 0.001.

To investigate the downstream molecular mechanism by which Sez6l regulates NP, we focused on the Wnt5a/Ca^2+^ non-canonical signaling pathway, which is known to mediate DRG neuronal sensitization and be regulated by Sez6l family proteins. Accumulating evidence has shown that the Wnt signaling pathway is overactivated in a wide variety of physiological and pathological processes. However, the functional roles of the Wnt signaling pathway in NP have not been fully clarified. Western blot results showed that the expression of Wnt5a, Frizzled2, and the ratio of p-CaMKII were significantly increased in SNI mice compared with the sham group (Figure 6A). The concentrations of IL-6, TNF-α, and IL-1β were reduced in SNI DRGs transfected with sh-Wnt5a (Figures 6B,C), whereas they remained unchanged in sham DRGs (Supplementary Figures S3D-SF). PWL and PWT assays also showed increased paw withdrawal latency and paw withdrawal threshold after sh-Wnt5a transfection (Figures 6E,F). Collectively, abnormal activation of the Wnt5a/Ca^2+^ signaling pathway promoted NP in the SNI model.

### Downregulation of Sez6l inhibited Wnt5a/Ca^2+^ signaling pathway and alleviates NP

**FIGURE 7:**
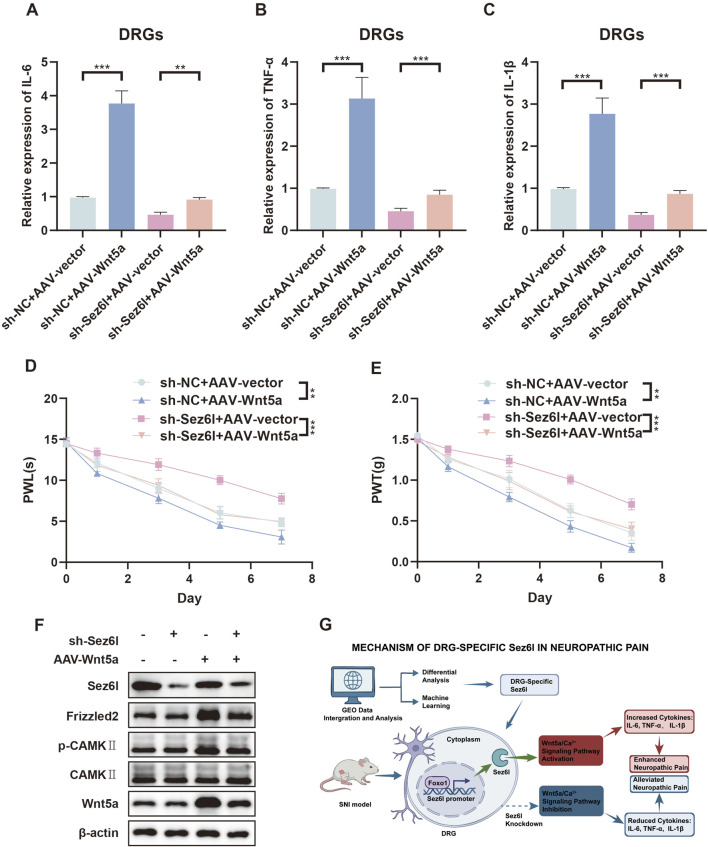
Effect of Sez6l knockdown on Wnt5a, Frizzled2, p-CAMKII expression and inflammatory cytokine levels in SNI mice DRGs. **(A–C)** ELISA analysis of IL-6, TNF-α, and IL-1β expression in DRGs from indicated mice. **(D,E)** PWL and PWT analysis of paw withdrawal latencies and threshold in indicated groups. **(F)** Western blot analysis of Wnt5a, Frizzled2, and p-CAMKII expression in DRGs from SNI mice after knockdown of Sez6l. **(G)** Schematic summarization of key findings presented in this study. n = 3 biological replicates per group for ELISA. n = 4 biological replicates per group for behavioral assays. Statistical analysis was performed using one-way ANOVA with Tukey’s post hoc test for ELISA and Western blot data, and repeated measures two-way ANOVA for behavioral data. Data are presented as mean ± SEM. *p < 0.05, **p < 0.01, ***p < 0.001.

We further investigated whether Sez6l regulated the Wnt5a/Ca^2+^ signaling pathway. ELISA results showed that the effect of silencing Sez6l on the expression of IL-6, TNF-α, and IL-1β was reversed by co-transfecting sh-Sez6l and Wnt5a overexpression vectors in DRGs of SNI mice (Figures 7A–C). PWL and PWT tests also showed that hyperalgesia and allodynia were attenuated by Sez6l knockdown but rescued by co-transfection of sh-Sez6l and Wnt5a overexpression vectors (Figures 7D,E). Western blot results showed that knockdown of Sez6l reduced the protein expression levels of Wnt5a, Frizzled2, and p-CaMKII (Figure 7F). In summary, Sez6l promoted NP through regulating the Wnt5a/Ca2+ signaling pathway (Figure 7G).

## Discussion

NP arises from functional abnormalities or structural damage within the nervous system itself, clinically characterized by hyperalgesia and allodynia (Finnerup et al., 2020). With global population aging and the escalating burden of chronic diseases, NP has emerged as a particularly refractory form of chronic pain that profoundly compromises patients’ quality of life (B et al., 2023). The pathogenesis of NP is notably complex, involving intricate interplay among neuroinflammation, epigenetic regulation, and ion channel dysfunction at multiple hierarchical levels (Basbaum et al., 2009). The DRG serve as the critical “first relay station” for sensory signal transmission and constitute a central hub in the initiation and maintenance of NP (Berta et al., 2023). The present study suggests a mechanistic link between Sez6l expression in DRG neurons and NP development through the activation of the Wnt5a/Ca^2+^ signaling pathway (Ong-Pålsson et al., 2021; Zhou et al., 2021). These findings provide novel insights into the molecular underpinnings of NP, specifically highlighting Sez6l as a previously unrecognized contributor to pain pathophysiology.

Machine learning has emerged as a transformative methodology for deciphering complex biological challenges across diverse domains, including healthcare, finance, and environmental science (Jordan and Mitchell, 2015; Topol, 2019). Distinguished by its capacity to process high-dimensional datasets, extract biologically meaningful patterns, and uncover non-obvious associations, machine learning techniques have been increasingly integrated into NP research and clinical management (Eraslan et al., 2019; Ma et al., 2014). Notable applications include: multivariate approaches to delineate distinct patterns of dynamic functional connectivity associated with NP (Cheng et al., 2018); unsupervised and supervised analyses to identify serum proteomic signatures linked to neuroinflammatory processes in breast cancer-related NP; and differential expression analysis combined with advanced machine learning algorithms to prioritize candidate genes from transcriptomic data. In this study, we employed differential analysis coupled with machine learning-based feature selection to identify Sez6l as a candidate gene of interest in DRG tissue. While the sample size (6 SNI DRG samples *versus* 10 sham controls) is relatively modest, the robust overexpression of Sez6l in the SNI model underscores its potential involvement in NP pathogenesis and justifies its prioritization for mechanistic investigation.

The Sez6l gene is widely expressed across mammalian tissues, with particularly enriched expression in the central and peripheral nervous systems (Ong-Pålsson et al., 2021). Existing literature has primarily implicated Sez6l in synaptic development and plasticity: the encoded protein participates in synapse formation and functional modulation, potentially influencing learning and memory through regulation of synaptic plasticity and neurotransmission (Pigoni et al., 2016). Consistent with this, Sez6l knockout mice exhibit marked deficits in water maze performance and display elevated anxiety-like and depressive-like behaviors in open field tests. Additionally, emerging evidence suggests Sez6l involvement in neurodegenerative pathologies, particularly Alzheimer’s disease (Nash et al., 2020). However, despite these advances, the specific molecular mechanisms of Sez6l and its functional roles under diverse neuropathological conditions—most notably in the context of chronic pain—remain largely unexplored (Roitman et al., 2019). Our study addresses this critical gap by demonstrating that Sez6l inhibition significantly attenuates NP-related behaviors and reduces the expression of key pro-inflammatory cytokines (IL-6, TNF-α, and IL-1β) in DRG tissue. These findings suggest a potential mechanistic association between Sez6l activity and neuroinflammation-mediated pain sensitization, positioning Sez6l as a novel molecular player in NP pathophysiology.

Transcription factors orchestrate complex gene regulatory networks underlying diverse physiological and pathological processes, including NP (Dou et al., 2023). Foxo1, the founding and most extensively characterized member of the FoxO family, governs critical cellular processes including cell cycle regulation, metabolic homeostasis, oxidative stress response, and apoptosis, with documented implications in immune disorders and tumorigenesis (Lu and Huang, 2011). Previous investigations have revealed functional interactions between Foxo1 and voltage-gated sodium channel Nav1.7 in the modulation of static mechanical pain (Zhang et al., 2021). Nevertheless, the specific roles of Foxo1 in NP pathogenesis remain poorly characterized. Through integrated bioinformatics analysis and ChIP validation, our study demonstrates that Foxo1 directly enhances Sez6l transcriptional activity, thereby establishing a novel Foxo1-Sez6l regulatory axis. To our knowledge, this study provides evidence for a transcriptional mechanism governing Sez6l expression in the context of NP.

Mechanistically, our study reveals that Sez6l promotes NP development, at least in part, through activation of the non-canonical Wnt5a/Ca^2+^ signaling cascade. The Wnt5a/Ca^2+^ pathway is recognized for its involvement in diverse cellular processes, including inflammatory responses and nociceptive signaling (Tang et al., 2022). Previous studies have established Wnt5a as a critical mediator in both neuropathic and inflammatory pain models (Simonetti and Kuner, 2020). Specifically, Lu et al. reported upregulated Wnt5a expression contributing to NP maintenance in the SNI model (Lu et al., 2022). However, the upstream regulatory mechanisms initiating Wnt5a/Ca^2+^ pathway activation in NP have remained elusive. Our findings demonstrate that Wnt5a, its receptor Frizzled2, and downstream effector p-CaMKII are markedly upregulated in DRGs of SNI mice, and that Sez6l overexpression significantly potentiates Wnt5a expression. These results position Sez6l as a candidate upstream regulator of the Wnt5a/Ca^2+^ cascade, thereby filling a critical gap in our understanding of NP molecular pathogenesis.

In conclusion, this study establishes Sez6l as a critical molecular determinant in the pathogenesis of SNI-induced NP. The convergence of three key findings—(i) significant Sez6l overexpression in DRG neurons, (ii) transcriptional upregulation by Foxo1, and (iii) functional coupling to Wnt5a/Ca^2+^ pathway activation. Several important questions warrant investigation in future studies: (i) validation of the Sez6l in human NP tissues and clinical cohorts; (ii) exploration of cell-type-specific roles of Sez6l in distinct DRG neuronal subpopulations; (iii) investigation of potential crosstalk between Sez6l-mediated signaling and other established NP mechanisms. By advancing our understanding of Sez6l biology in pain processing, this work lays the groundwork for mechanism-based therapeutic development that may ultimately translate into improved quality of life for patients suffering from refractory NP.

## Conclusion

In summary, Foxo1-induced Sez6l was specifically upregulated in DRGs of the SNI model. Sez6l inhibition relieved hyperalgesia and allodynia and reduced the expression of IL-6, TNF-α, and IL-1β. Mechanistically, Sez6l promoted the expression of Wnt5a, Frizzled2, and p-CaMKII, potentially contributing to NP pathogenesis.

## Data Availability

The original contributions presented in the study are publicly available. This data can be found at the Gene Expression Omnibus repository with the accession numbers GSE89224 and GSE102721.

## Supplementary material

SUPPLEMENTARY FIGURE S1
**(A)** Western blot revealed that Sez6l expression was significantly upregulated in the DRGs of SNI mice. **(B)** qRT-PCR assays was applied to evaluate the expression of Sez6l, Cacna2d1 and Atf3. **(C,D)** ELISA analysis of IL-6, TNF-α, and IL-1β expression in the DRGs. n = 3 biological replicates per group for ELISA, qRT-PCR and Western blot. Statistical analysis was performed using one-way ANOVA with Tukey’s post hoc test or unpaired two-tailed Student’s t-test as appropriate. Data are presented as mean ± SEM. *p < 0.05, **p < 0.01, ***p < 0.001.

SUPPLEMENTARY FIGURE S2
**(A)** qRT-PCR assay was applied to evaluate the expression of Foxo1, Foxo3, Vdr and SOX10 in Sham or SNI DRGs. **(B–E)** qRT-PCR results indicated that the Foxo1, Foxo3, Vdr and SOX10 expression was decreased after transfection of indicated shRNAs. **(F,G)** Western blot analysis of Sez6l expression in indicated groups. n = 3 biological replicates per group for qRT-PCR and Western blot. Statistical analysis was performed using one-way ANOVA with Tukey’s post hoc test or unpaired two-tailed Student’s t-test as appropriate. Data are presented as mean ± SEM. *p < 0.05, **p < 0.01, ***p < 0.001.

SUPPLEMENTARY FIGURE S3
**(A–C)** ELISA assay results showing the expression levels of IL-6, TNF-α, and IL-1β in SNI DRGs transfected with sh-Foxo1 and Sez6l overexpressing vectors. **(D–F)** ELISA assay analysis of IL-6, TNF-α, and IL-1β expression in the indicated Sham DRGs. n = 3 biological replicates per group for ELISA. Statistical analysis was performed using one-way ANOVA with Tukey’s post hoc test. Data are presented as mean ± SEM. *p < 0.05, **p < 0.01, ***p < 0.001.
