# Supplementary figures and images for: Sez6l promotes neuropathic pain via Wnt5a/Ca2+ pathways in dorsal root ganglion
Source: Front Genet. 2026 Apr 20;17:1799301. doi: 10.3389/fgene.2026.1799301 (PMC13135866; doi:10.3389/fgene.2026.1799301)

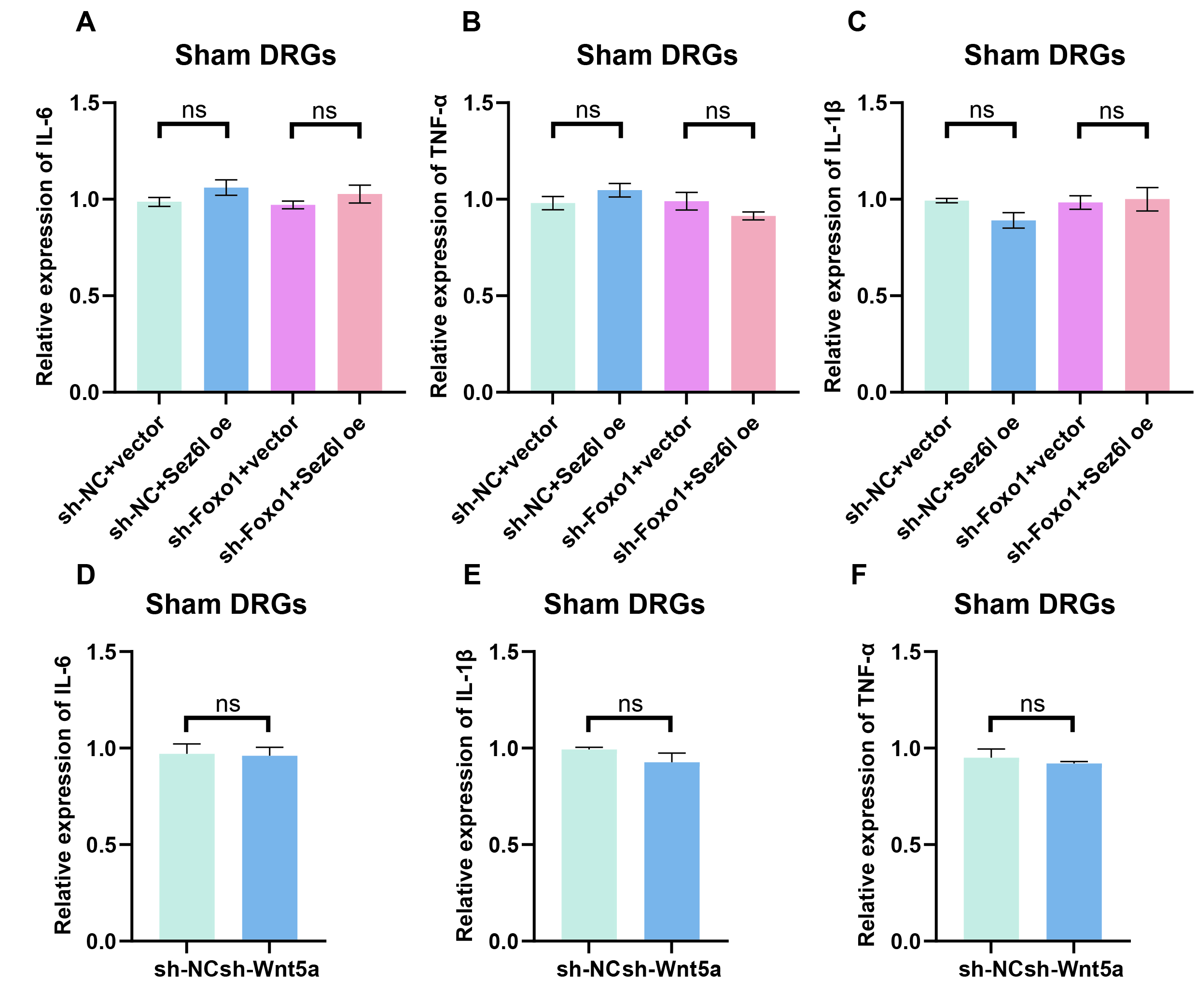

Supplement: Supplementary file 1 [file Image3.jpg]

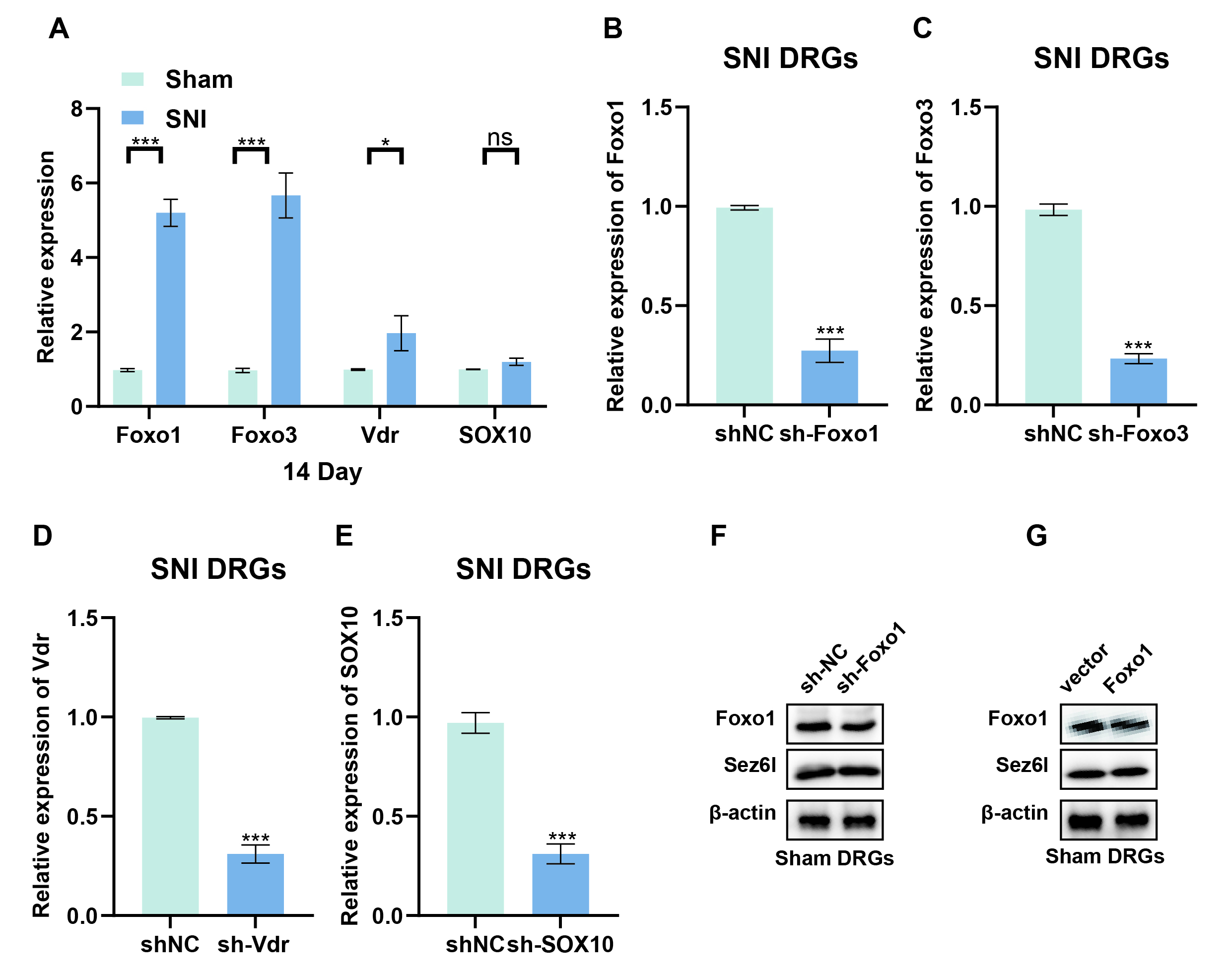

Supplement: Supplementary file 2 [file Image2.jpg]

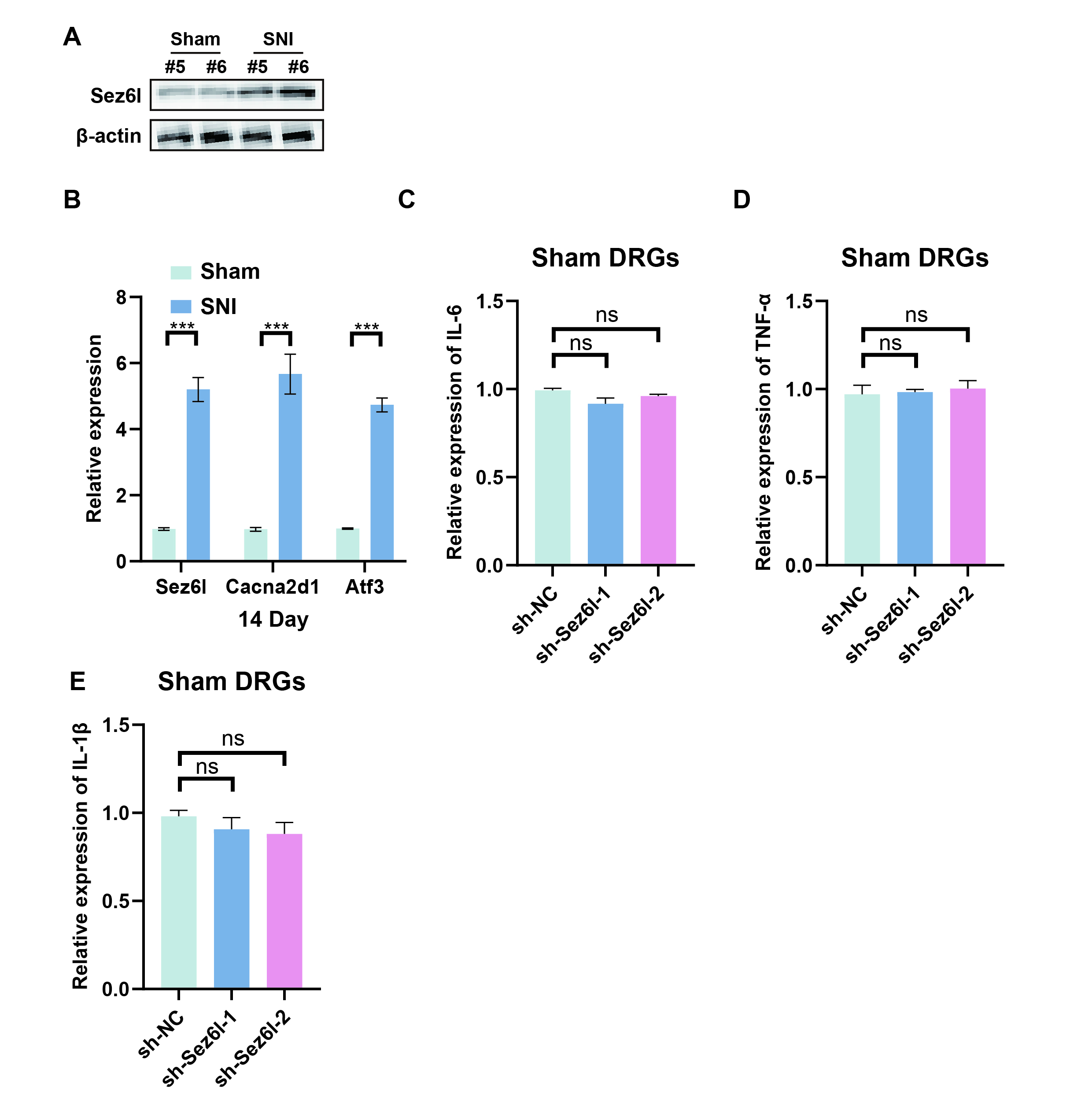

Supplement: Supplementary file 3 [file Image1.jpg]
